# Reduced Liver Autophagy in High-Fat Diet Induced Liver Steatosis in New Zealand Obese Mice

**DOI:** 10.3390/antiox10040501

**Published:** 2021-03-24

**Authors:** Ioanna Korovila, Annika Höhn, Tobias Jung, Tilman Grune, Christiane Ott

**Affiliations:** 1 Department of Molecular Toxicology, German Institute of Human Nutrition, Potsdam-Rehbruecke, 14558 Nuthetal, Germany; Ioanna.Korovila@dife.de (I.K.); Annika.Hoehn@dife.de (A.H.); Tobias.Jung@dife.de (T.J.); Christiane.Ott@dife.de (C.O.); 2 German Center for Diabetes Research (DZD), 85764 München-Neuherberg, Germany; 3 German Center for Cardiovascular Research (DZHK—Partner Site Berlin), 10117 Berlin, Germany; 4 Department of Physiological Chemistry, Faculty of Chemistry, University of Vienna, 1090 Vienna, Austria; 5 Institute of Nutritional Sciences, University of Potsdam, 14558 Nuthetal, Germany

**Keywords:** proteostasis, protein modification, 4-HNE, proteasome, lipid droplets

## Abstract

Non-alcoholic fatty liver disease (NAFLD), as a consequence of overnutrition caused by high-calorie diets, results in obesity and disturbed lipid homeostasis leading to hepatic lipid droplet formation. Lipid droplets can impair hepatocellular function; therefore, it is of utmost importance to degrade these cellular structures. This requires the normal function of the autophagic-lysosomal system and the ubiquitin-proteasomal system. We demonstrated in NZO mice, a polygenic model of obesity, which were compared to C57BL/6J (B6) mice, that a high-fat diet leads to obesity and accumulation of lipid droplets in the liver. This was accompanied by a loss of autophagy efficiency whereas the activity of lysosomal proteases and the 20S proteasome remained unaffected. The disturbance of cellular protein homeostasis was further demonstrated by the accumulation of 3-nitrotyrosine and 4-hydroxynonenal modified proteins, which are normally prone to degradation. Therefore, we conclude that fat accumulation in the liver due to a high-fat diet is associated with a failure of autophagy and leads to the disturbance of proteostasis. This might further contribute to lipid droplet stabilization and accumulation.

## 1. Introduction

Overnutrition as a result of high-calorie diets induces obesity, metabolic stress, insulin resistance and beta-cell failure [1]. One major health problem associated with diet-induced obesity is non-alcoholic fatty liver disease (NAFLD) as a result of disturbed lipid homeostasis and increased lipid accumulation in the liver [2], leading to hepatocellular dysfunction. The phenomenon of lipotoxicity plays a significant role in the pathogenesis of liver failure [3], even if the exact mechanism remains to be elucidated. Excess lipids are stored in lipid droplets, organelles containing a core of neutral lipids surrounded by a phospholipid bilayer, and regulated by inclusion proteins [4]. Perilipins are lipid droplet-associated proteins and in NAFLD perilipin 2 (plin2) is the dominant form [2,5], exerting a multitude of functions in hepatocytes [4,5,6,7]. Autophagy, particularly lipophagy, has been found to be responsible for the degradation of bulky amounts of lipid droplets. The autophagy-lysosomal system (ALS) is a complex degradation machinery involving more than 30 autophagy-related (Atg) proteins, including several regulatory and lysosomal membrane proteins as well as hydrolases [8]. Degradation is often limited by substrate uptake via autophagy rather than by the efficiency of lysosomal degradation [8]. Proteins such as the microtubule-associated proteins 1A/1B light chain 3B (LC3-I/LC3-II), Atg5, Atg5-Atg12 complex, and p62 are proteins that monitor autophagic activity [8,9]. However, levels of these proteins must be interpreted with caution, as they may be the result of reduced protein expression or increased autophagy flux [10].

On the other hand, the ubiquitin-proteasomal system (UPS) comprises the ubiquitination targeting machinery and final proteasomal degradation [11,12]. While the 20S proteasome, the catalytic core of the UPS, is responsible for the degradation of slightly to moderately oxidized and unfolded proteins, the 26S proteasome (a 20S proteasome capped with the 19S regulator complex) degrades ubiquitin-tagged proteins in an ATP-dependent manner. However, the 20S proteasome is the core of the UPS and therefore, it is responsible for the catalytic degradation [11].

Decreased degradation capacity or increased oxidative stress due to a high-fat diet can disturb the balance in proteostasis, which leads to the accumulation of modified proteins and aggregates. Two markers of lipoxidative stress and protein damage are protein-bound 3-nitrotyrosine (3-NT) [13] and 4-hydroxynonenal (4-HNE) [14], which play a causal role in lipoxidation-derived damage and accelerated aging [15].

However, the role of high-fat diet (HFD) and lipid droplet accumulation in the activity of the proteolytic systems in the liver remains obscure, although several mechanisms have been proposed [16,17]. Therefore, we investigated the effect of prolonged high-fat diet-induced lipid accumulation on the activity of proteolytic systems in the liver of New Zealand obese mice (NZO), which develop severe obesity, compared to the widely used standard C57BL/6J (B6) mouse as a normal, healthy wildtype control.

## 2. Methods

### 2.1. Animal Experimental Procedure

All animal procedures were performed in accordance with the guidelines of the German Law on the Protection of Animals and the experimental protocol was reviewed and approved by the local authorities (Landesamt für Arbeitsschutz, Verbraucherschutz und Gesundheit Brandenburg, Germany, Brandenburg, approval number: V3-2347-21-2015). The animal experiments are partially described in [18]. Briefly, male C57Bl/6J and male NZO mice (C57Bl/6J (B6) and NZO/HIBomDIfE, German Institute of Human Nutrition, Potsdam-Rehbruecke, Germany) were housed in open cages of 4–5 animals in a controlled environment (20 ± 2 °C, 12/12 h light/dark cycle) with ad libitum access to diet and water. Seven-week old mice received a standard diet (SD; V1534-300 Ssniff, Soest, Germany) or a carbohydrate-free, high-fat diet (HFD, #C105789, Altromin, Lage, Germany) for 15 or 32 weeks. Mice were sacrificed by acute isoflurane exposure and blood/tissue samples were collected. Tissue was collected for either histological analysis, samples were fixed in 4% para-formaldehyde or molecular biological analysis, and immediately frozen by liquid nitrogen. Body and liver weight were measured with an electronic scale. The triglyceride (TG) content in liver tissues in HB buffer (10 mM NaH_2_PO_4_·H_2_O, 1 mM EDTA, pH 7.4, 1% Polyoxyethylene (10) tridecyl ether) was quantitatively determined by using ABX Pentra Triglycerides CP kit (Horiba; A11A01640, Axon Lab AG, Stuttgart, Germany) on an autoanalyzer Cobas mira (Roche).

### 2.2. Immunoblotting

Liver tissue was lysed with HB buffer and homogenized. The lysate was subsequently centrifugated for 30 min (23,100× *g*, 4 °C). The supernatant was collected and centrifuged. The supernatant was collected and stored at −20 °C, while a part was used to determine protein concentration by the Lowry protein assay.

Proteins were separated by a 10% or 15% SDS-PAGE gel electrophoresis and transferred to nitrocellulose membrane by semi-dry blotting and detected by indirect fluorescence through the Odyssey imaging system. Procedures were performed according to the manufacturer’s instructions. As primary antibodies were used: perilipin 2 (R&D Systems; MAB7634); Atg5 (nanotools; #0262), p62 (abcam; ab56416), LAMP1 (cell signaling; #3243), LC3A/B (cell signaling; #12741S), 3-nitrotyrosine (abcam, ab110282), 4-hydroxynonenal (abcam, ab46545). The membranes were then probed with fluorescent-labeled secondary antibodies. Both primary and secondary antibodies were diluted in LI-COR Odyssey Blocking Buffer/PBS (1:2) containing 0.1% Tween-20. Detection and quantification of immunoblots were performed in a linear range with the LI-COR Odyssey^®^ imaging system. Proteins were normalized to total protein amount via Ponceau S staining.

### 2.3. Immunohistochemistry: H/E Staining 

Liver slides (paraffin sections 2µm) were deparaffinized using Roti^®^-Histol (Carl Roth, 6640) and rehydrated by ethanol gradient (100–40%). H&E staining was performed by firstly adding hematoxylin solution (Sigma-Aldrich, GHS316, MerckKGaA, Darmstadt, Deutschland) for 45 s followed by 10 s tap water and incubation of eosin (Sigma-Aldrich, HT110232, MerckKGaA, Darmstadt, Deutschland) for 1 min. After staining, samples were mounted with Entellan^®^ (VWR, 1079610500, MerckKGaA, Darmstadt, Deutschland). Liver sections were scanned using a MIRAX Scanner from Zeiss and software MIRAX Viewer. 

### 2.4. Proteasomal Activity

For maximum proteasome activity, liver tissue samples were homogenized with a tissue lyser (Qiagen, Hilden, Germany) in lysis buffer (250 mM sucrose, 25 mM HEPES, 1 mM EDTA, 10 mM magnesium chloride and freshly added 1.7 mM DTT, pH 7.8), followed by passing lysates through a 27-gauge needle, freeze-thaw cycles and centrifugation at 13,400× *g* rpm, for 10 min, at 4 °C. Supernatants were used for protein determination (Bradford assay) and proteasome activity assay. Samples were adjusted to 1 mg/mL protein and incubated with proteasome incubation buffer (containing 225 mM Tris buffer (pH 7.8), 7.5 mM magnesium acetate, 45 mM potassium chloride, 7.5 mM magnesium chloride and freshly added 1 mM DTT). To measure 20S proteasome activity, ATP was depleted by adding 15 mM 2-deoxyglucose and 0.1 mg/mL hexokinase to the incubation buffer. Chymotrypsin-like proteasome activity was measured using fluorogenic peptide suc-Leu-Leu-Val-Tyr-7-AMC (Enzo, #BML-P802-0005, final concentration 166 µM/well). AMC liberation was determined in a black 96-well plate at 37 °C using a fluorescence microplate plate reader (excitation: 360 nm, emission: 460 nm). Proteolytic activity was calculated using free 7-amino-4-methylcoumarin (AMC) as the fluorogenic calibration standard and verified using proteasome inhibitor lactacystin (Enzo Life sciences GmbH, BML-PI104-1000, Loerrach, Germany).

### 2.5. Lysosomal Activity

For measuring the lysosomal activity, here, the cysteine cathepsin activity, liver tissue was homogenized in 500 µL of 1 mM DTT/PBS, shaken for 1 h at 4 °C, sonicated on ice for 2 min at 50% amplitude and subsequently centrifuged for 20 min at 14,000 rpm. Further, 10 µg of lysates were incubated with lysosome incubation buffer (containing 24 mM L-Cysteine hydrochloride (L-Cys-OH HCl), 150 mM Na-Acetate, 3 mM EDTA Dihydrate, pH 4.0) for 10 min. To measure cysteine cathepsin activity, OmniCathepsin fluorogenic substrate Z-FR-AMC, Z-Phe-Arg-AMC (Enzo #BML-p-139) was used, with a final substrate concentration of 166 µM, to determine AMC liberation, which was monitored every 3 min for 90 min using a fluorescence microplate reader (excitation: 360 nm, emission: 460 nm). Proteolytic cathepsin activity was calculated using free 7-amino-4-methylcoumarin (AMC) as the fluorogenic calibration standard and proteolytic activity was verified using a protease inhibitor cocktail (Sigma-Aldrich, P8340, diluted according to manufacturer’s instructions).

### 2.6. Statistics

Statistical analysis was performed using GraphPad Prism (GraphPad Software, San Diego, USA; v. 8.0.0). Initially we tested for normal distribution using a Shapiro–Wilk or Kolmogorov–Smirnov test. If the values were normally distributed, either two-way-ANOVA, comparing NZO diet and age (samples shown after the dashed line in the figures below), unpaired t-test (indicated with *) or one sample t-test (indicated with #), comparing two selected samples, were applied. Statistical significance was considered and indicated at *p* < 0.05 and results are presented as mean values ± standard deviation.

## 3. Results

**Figure 1:**
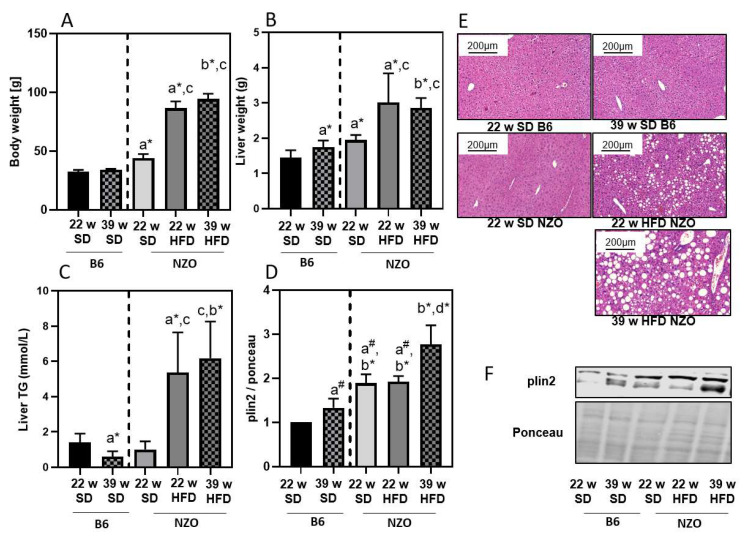
High-fat diet leads to enhanced liver lipid accumulation in NZO mice. B6 and NZO mice were fed by a standard diet (SD) or a high-fat diet (HFD) until the indicated age of 22 or 39 weeks. Body weight (panel **A**), liver weight (panel **B**), triglycerides (TG) (panel **C**) and plin2 content (panel **D**) were measured as described in the Methods section. Plin2 (panel **D**) was determined by immunoblotting and data were normalized towards 22w SD B6 (set as ‘1’). A representative blot is shown in panel **F**. Representative H&E staining of liver slices to visualize the lipid droplets are shown in panel **E**. Statistical analysis was performed either by two-way ANOVA comparing NZO mice by diet, unpaired *t*-test (indicated by *) or one sample *t*-test (indicated by #), comparing selected samples directly. Statistical significance means: a—versus 22w B6; b—vs. 39w B6; c—vs. 22w Nzo SD; and d—vs. 22w NZO HFD. The data presented are the mean ± standard deviation, *n* = 5–8.

NZO mice are a well-established model to study type II diabetes and obesity [18]. A number of different feeding strategies are known to induce an obesity-related phenotype in this mouse strain, even though these mice already gain weight on a standard diet (SD). To better understand and interpret the results in the NZO mice, we additionally added data for B6 mice as a normal, healthy wildtype control. Compared to C57BL/6J (B6), NZO mice gained weight on a SD at 22 weeks of age, while the HFD resulted in them being severely overweight during the same feeding period. (Figure 1A). Continuation of high-fat feeding in NZO mice resulted in further, but less pronounced weight gain (Figure 1A). Weight gain was accompanied by an increase in liver weight until week 22 in NZO mice (Figure 1B). In addition, we measured liver TGs and plin2, a major lipid droplet protein (Figure 1C,D,F). Both parameters revealed an enhanced lipid accumulation. This could be observed by H&E staining of the liver, which showed an increase in lipid droplet size and content (Figure 1E).

**Figure 2:**
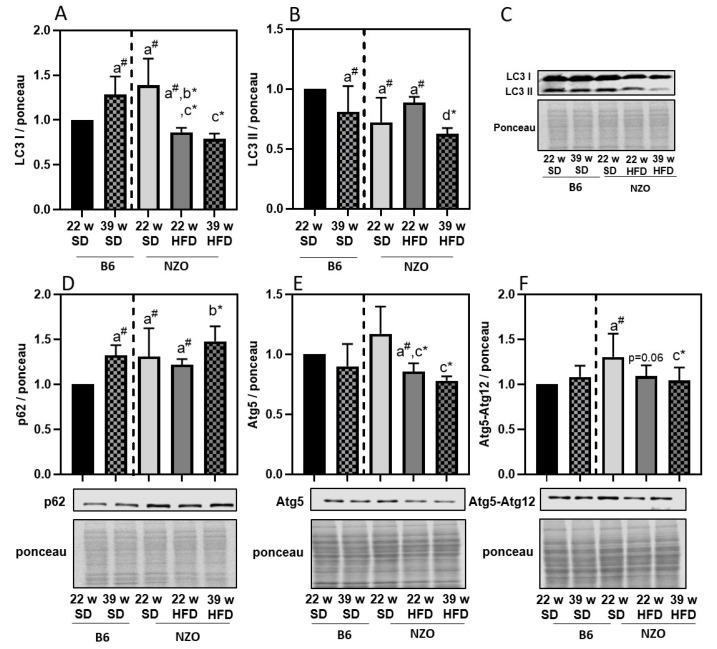
Autophagy-related protein expression in liver of high-fat diet exposed mice. B6 and NZO mice were fed by a standard diet (SD) or a high-fat diet (HFD) as described above. Panel **A** and panel **B** shows LC3-I and LC3-II protein amount, whereas p62 (panel **D**), Atg5 (panel **E**) and Atg5-Atg12 (panel **F**) are presented together with respective immunoblots and ponceau staining. Panel C is showing a representative LC3 staining with the relative ponceau staining. All data were normalized towards 22w SD B6 (set as ‘1’). Statistical analysis was performed by one sample *t*-test (indicated by #) or unpaired *t*-test (indicated by *), directly comparing two selected samples. Statistical significance means: a—versus 22w B6; b—vs. 39w B6; c—vs. 22w Nzo SD; and d—vs. 22w NZO HFD. The data presented are the mean ± standard deviation, *n* = 5–8.

Since our aim was to investigate the effects of lipid droplet accumulation on liver proteostasis, we next determined the parameters of the ALS and UPS. Regarding the ALS, we initially analyzed the relative protein expression of autophagy-related proteins *SQSTM1* (p62), Atg5, Atg5-Atg12 as well as LC3-I and LC3-II in the liver tissue. As demonstrated in Figure 2, LC3-I and Atg5 were decreasing in NZO mice on HFD, compared to NZO on SD. LC3-II levels of NZO SD are lower compared to B6 SD. Furthermore, LC3-II expression was lower in 39w NZO HFD compared to 22w NZO HFD mice. The p62 protein expression was increased in 39w B6 mice and is higher in NZO mice compared to the 22w B6. Prolonged HFD slightly enhanced p62 protein in the 39w NZO HFD, compared to 22w NZO HDF and 39w B6 SD, indicating that p62 turnover might be reduced over time and by HFD (Figure 2D). Autophagy-related protein Atg5-Atg12 tends to decline in 22w NZO HFD compared to 22w NZO SD, but were more distinctly decreased by prolonged HFD in NZOs (Figure 2E). Since Atg5 and Atg5-Atg12 conjugate play an important role in the lipidation of LC3-I to LC3-II, reduced levels of both would support the assumption of reduced autophagy in the liver tissue of high-fat fed mice, which further contributes to lipid droplet accumulation.

**Figure 3:**
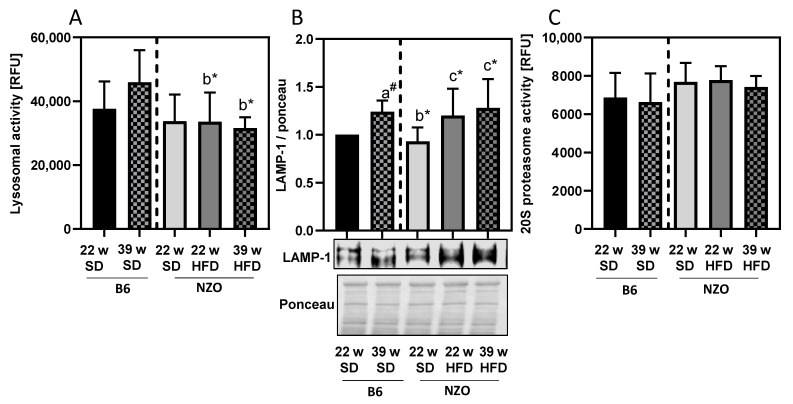
Protein degradation systems in liver of high-fat diet exposed mice. Lysosomal (panel **A**) and 20S proteasomal (panel **C**) activities were determined as described in the Methods section. Panel **B** shows the amount of the LAMP-1 protein together with a representative blot and the corresponding ponceau staining. Data in Panel A and B were normalized towards 22w SD B6 (set as ‘1’). Statistical analysis was performed by one sample *t*-test (indicated by #) or unpaired *t*-test (indicated by *), directly comparing two selected samples. Statistical significance means: a—versus 22w; b—vs. 39w B6 and c—vs. 22w NZO SD. The data presented are the mean ± standard deviation, *n* = 5–8.

Besides analysis of autophagy proteins, we also analyzed lysosomal enzyme activity to estimate changes in the ALS. Thus, we measured the maximal cysteine cathepsin activity in the liver tissue of all groups (Figure 3A). Interestingly, the activity of the lysosomal cysteine proteases seems to be generally lower in the NZO mice compared to B6 mice, but was unaffected by HFD (Figure 3A) whereas the content of lysosomes, quantified via LAMP1 protein expression, seems to increase as a compensatory measure (Figure 3B). So, it seems that the limiting process in the ALS is declining autophagy rather than lysosomal degradation. Furthermore, we tested the 20S proteasome activity as the catalytic core of the UPS, but could not detect any changes within the groups (Figure 3C).

**Figure 4:**
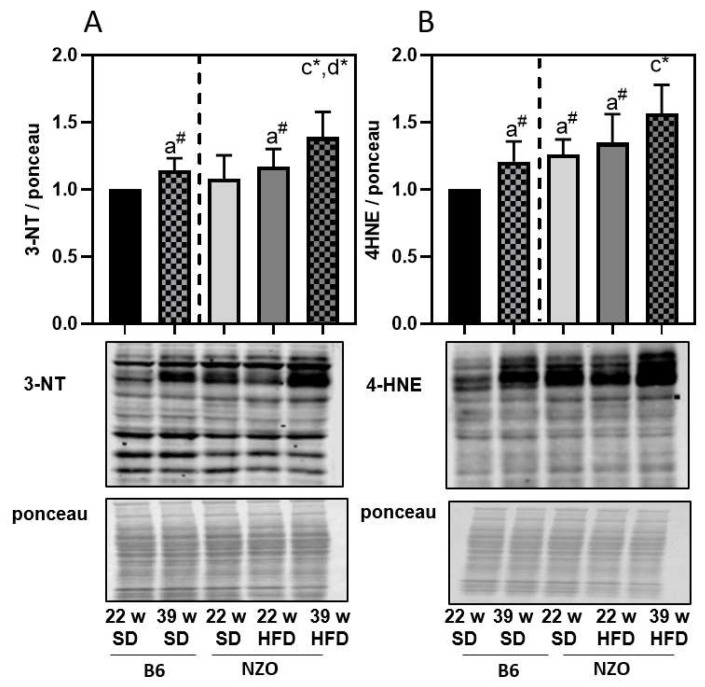
Nonenzymatic protein modification in liver of high-fat diet exposed mice. Protein-bound 3-nitrotyrosine (panel **A**) and 4-hydroxynonenal-protein modification (panel **B**) were determined by immunoblotting as described in the methods section. Each panel shows a representative blot and the corresponding ponceau staining. All data were normalized towards 22w SD B6 (set as ‘1’). Statistical analysis was performed by one sample *t*-test (indicated by #) or unpaired *t*-test (indicated by *), directly comparing two selected samples. Statistical significance means: a—versus 22w B6; c—vs. 22w NZO SD; and d—vs 22w NZO HFD. The data presented are the mean ± standard deviation, *n* = 5–8.

Disturbances in protein degradation, starting here in the ALS, can consequently lead to an accumulation of modified proteins, so we also measured the amounts of modified proteins in liver samples. Therefore, we analyzed the amount of nitrated (Figure 4A) and 4-HNE-modified proteins (Figure 4B). Quantification of accumulated modified proteins revealed a clear dependence on long-term high-fat feeding (Figure 4), indicating that lipid droplet formation and decline in the ALS contributes to a general disbalance of proteostasis in the liver.

## 4. Discussion

Metabolic syndrome and type II diabetes are associated with a number of complications including liver steatosis, which may lead to NAFLD [2]. Accumulation of lipids in the liver might have serious effects on the metabolic performance of hepatocytes [19]. To investigate the impact of obesity and high-fat feeding on proteostasis in the liver, we used NZO mice, an established model for polygenic obesity. NZO mice gain weight on a standard diet compared to wildtype B6 mice, exhibit obesity and develop insulin resistance when fed a HFD. However, if challenged with nutritional carbohydrates, a progressive loss of beta-cells can be observed [18,20,21,22,23,24]. In our experiments 22w NZO SD mice already showed a higher increase in body and liver weight as well as plin2 content, compared to 22w B6 on SD. These parameters are further increased if NZO are fed a HFD. In addition, a prolonged HFD resulted in a gradual increase in lipid droplets and liver TGs. This is in accordance with the work by Nocetti et al. [25], demonstrating the accumulation of fat in lipid droplets. While it is generally assumed that the accumulation of lipid droplets in liver is due to exposure to high nutritional fat [26], it can be expected that the liver has some capacity to prevent unwanted lipid droplet accumulation by initiating the transport of TGs to the adipose tissue. This would require a functional breakdown of lipid droplets, among others, mediated either by the UPS or the ALS [27,28]. Autophagy has been shown to contribute to the cellular energy balance, providing free amino and fatty acids as energetically essential components. However, cells not only activate lipolysis when they need energy but also to prevent stores from becoming enlarged [29]. Although, mobilization of lipid droplets by lipolysis has been attributed to cytosolic lipases, other studies have reported a role for autophagy (lipophagy) [30]. A study in hepatocytes knocked down for Atg5 provided the first evidence that lipid droplets are a substrate for autophagy [31]. Furthermore, Atg7 knockout in liver led to accelerated development of liver steatosis [32].

Our data suggest that a decrease in early autophagy initiation proteins is a starting point for the decline in autophagy that is associated with increasing amounts of lipid droplets and TGs in liver tissue of high-fat fed mice. It has already been demonstrated that high fat conditions can time-dependently impair autophagy in liver cells by palmitate treatment of HepG2 cells [33]. In addition to altered autophagy proteins, we also detected changes in maximal lysosomal activity.

While lysosomal cathepsin activity was generally lower in NZO mice, it was not affected by HFD. Interestingly, the lysosome content, measured by LAMP-1, increased in liver of NZO on HFD, indicating a compensatory upregulation of lysosomes. Furthermore, a study by Declèves et al., using electron microscopy, found that obese mice showed increased LAMP-1 levels and enlarged lysosomes in kidneys, suggesting an overload of the lysosomal system and accumulation of lysosomes [34].

On the contrary, the 20S proteasome activity was not changed. Since our data indicate an incipient decline of autophagy in the liver through HFD, we also considered an increase in modified proteins. We were able to show an accumulation of protein-bound 3-nitrotyrosine and 4-HNE modified proteins in the high-fat fed mice. This was also observed in B6 mice on a HFD by Gutiérrez-Camacho et al. [35], although additional challenges were applied in the study. Both protein modifications have been previously shown in HFD-induced stress [36,37] and aging [14,38,39]. Interestingly, similar levels of modified proteins were observed in liver tissue of 22w NZO on SD as in the older 39w B6 SD, which were additionally enhanced due to long-term HFD in 39w NZO HFD.

In conclusion, HFD feeding induces lipid droplet formation and leads to increased TG levels, which are associated with reduced autophagy and accumulation of modified proteins in the liver, indicating impaired protein turnover. Therefore, the activation of the autophagy pathway might lead to a reduction of lipid accumulation in the liver, as demonstrated in vitro by the use of punicalagin [33]. Another approach would be the reduction of liver fat by nutritional means, as shown for a high protein diet in humans [40], which might have effects on the autophagic flux in the liver, although the maximal autophagic capacity seems to be reduced. Furthermore, an interesting approach would be to increase the antioxidative defense mechanisms, which were also shown to result in a reduced load on oxidative damage [41].

Moreover, it appears that obese 22w NZO mice on SD show more similarities to the older 39w B6 mice, e.g., reduced autophagy and increased modified proteins, which are further enhanced by HFD, suggesting accelerated aging in obese mice and through HFD. To clarify whether HFD-reduced autophagy could lead to accelerated aging, further studies are required, including additional controls.

## Data Availability

The data used to support the findings of the present study are available from the corresponding author upon request.

## Abbreviations

ALS	Autophagy-Lysosomal System
AMC	7-amino-4-methylcoumarin
Atg	Autophagy-Related Gene
B6	C57BL/6J Mouse
HFD	High-Fat Diet
4-HNE	4-hydroxynonenal
LAMP	Lysosomal-Associated Membrane Protein
LC3	Microtubule-Associated Proteins Light Chain 3
NAFLD	Non-Alcoholic Fatty Liver Disease
NZO	New Zealand Obese
plin2	Perilipin 2
RNS/ROS	Reactive Nitrogen/Oxygen Species
SD	Standard Diet
UPS	Ubiquitin-Proteasomal System
